# Financial implications of unpaid clinical placements for allied health, dentistry, medical, and nursing students in Australia: a scoping review with recommendations for policy, research, and practice

**DOI:** 10.1186/s12913-024-11888-y

**Published:** 2024-11-15

**Authors:** Hannah Beks, Sandra Walsh, Suzanne Clayden, Lucinda Watson, Joyti Zwar, Laura Alston

**Affiliations:** 1 School of Medicine, Deakin Rural Health, Deakin University, PO Box 423, Warrnambool, VIC Australia; 2 University Department of Rural Health, University of South Australia, Whyalla, South Australia Australia; 3 Specialist Physicians Clinic, South West Healthcare, Warrnambool, VIC Australia; 4 School of Health and Social Development, Deakin University, Warrnambool, VIC Australia; 5 Colac Area Health, Colac, VIC Australia

**Keywords:** Students, Health occupations, Allied health, Dentistry, Medicine, Nursing, Clinical placements, Clinical clerkship

## Abstract

**Background:**

Investing in allied health, dentistry, medical, and nursing undergraduate and postgraduate qualifying education is critical to meet a growing demand on global health care systems. Clinical placements are an integral component of qualifying training and are conventionally unpaid. Widespread economic challenges, attributed to a post-COVID-19 pandemic recovery era and global unrest, have led to growing economic hardship for populations, even in high-income countries like Australia. Allied health, dentistry, medical, and nursing undergraduate and postgraduate students undertaking unpaid clinical placements are not immune from these stressors, which has implications for education providers, ageing populations, the future health care system, and policy-makers. The purpose of this review was to better understand these stressors by scoping the financial implications of unpaid clinical placements for allied health, dentistry, medical, and nursing students in Australian research.

**Methods:**

The Joanna Briggs Institute’s scoping review methodology was used. This involved a search of academic databases and an extensive search of grey literature sources. Literature published from 1 January 2014 was included. Citations were independently screened by two reviewers.

**Results:**

Thirty-three research studies were included. Most studies focused on allied health students (*n* = 12), followed by nursing (*n* = 11), and medical students (*n* = 5), with an additional five studies focused on multiple disciplines, including dentistry. One study had an interventional component. Findings were grouped around four concepts: reliance on self-reported measures of financial implications, costs of unpaid clinical placements for students, implications of costs for students, and an urgent need for targeted strategies to redress.

**Conclusions:**

The financial implications of unpaid clinical placements for allied health, dentistry, medical, and nursing students in Australia are well-established in research. Impacts are significant for the future of Australia’s health workforce and health system. Research findings have been consistent over the past decade in advocating for greater financial support for students undertaking unpaid clinical placements and flexibility of placement models to mitigate the indirect costs of placements. Collaboration between state and federal government, universities, peak professional bodies, and placement host organisations is imperative to implement a suite of strategies to redress the financial burden experienced by students and secure the future of Australia’s health workforce.

**Supplementary Information:**

The online version contains supplementary material available at 10.1186/s12913-024-11888-y.

## Introduction

Investing in allied health, dentistry, medical, and nursing undergraduate and postgraduate qualifying education is critical to meet a growing demand on global health care systems [1]. Clinical placements are an integral component of qualifying training [2]. There is a wealth of global research evidence describing clinical placement models for respective professions across a range of clinical and community contexts; including longitudinal integrated clerkships for medical students [3], rural placements for nursing students [4], and service-learning placements for allied health students (patient facing disciplines with a defined scope of practice and accreditation that are not nursing, medicine, or dentistry) [5, 6]. However, clinical placements undertaken as part of qualifying degrees, are conventionally unpaid; and tend to be full-time, limiting the capacity of students to maintain external paid employment that is required to fund living throughout their degree. The length of placements varies by discipline and training programs. An international review of clinical placements across disciplines found placement length to range from 6 days to 52 weeks, with a mean of 10 weeks [7].

Widespread economic challenges, attributed to a post-COVID-19 pandemic recovery era and global unrest, have led to growing economic hardship for populations, even in high-income countries [2]. In Australia, this has had flow on effects for households attributed to an elevated consumer price index (inflation), which has augmented financial stress, food insecurity, and homelessness [3–5]. Allied health, dentistry, medical, and nursing undergraduate and postgraduate students are not immune from these stressors and the health and wellbeing impacts, which has implications for education providers, ageing populations, the future health care system, and policy-makers. This includes promoting the recruitment of mature-age students to post-graduate health degrees, such as allied health and medicine [8]. Aside from some discussion in the Australian [9–11] media and advocacy of student groups (e.g., Students Against Placement Poverty), there has been little systematic examination of the financial implications of unpaid clinical placements from a student perspective, particularly considering global pressures. Consolidating knowledge on this issue is important to develop strategies to redress inequities experienced by students.

An initial search was undertaken in Ovid MEDLINE, Joanna Briggs Institute’s Evidence Synthesis, and PROSPERO for both existing reviews and proposed reviews. No reviews examining financial stress in nursing, allied health, dentistry, and medical students completing undergraduate or postgraduate qualifying degrees and undertaking unpaid clinical placements, were identified. A proposed international systematic review was identified that sought to identify sources of stress for undergraduate nursing students undertaking clinical placements but did not explicitly seek to report on financial implications [10].

The research question for this proposed systematic scoping review was:

What literature is available examining the financial implications of unpaid clinical placements for allied health, dentistry, medical, and nursing students in Australia?

Review objectives included:

1. To search for literature examining financial implications of unpaid clinical placements for allied health, dentistry, medical, and nursing students in Australia,
2. To scope how the financial implications of unpaid clinical placements for allied health, dentistry, medical, and nursing students in Australia are measured in research, and
3. To synthesise the key concepts related to the financial implications of unpaid clinical placements for allied health, dentistry, medical, and nursing students in Australia with implications for policy, research, and practice.

## Methods

The Joanna Briggs Institute’s (JBI) scoping review methodology was used [13]. The Preferred Reporting Items for Systematic Reviews and Meta-analysis extension for Scoping Reviews (PRISMA-ScR) [14] checklist was reported against (Supplementary file 2). Methods were specified in advance (Open Science Framework: doi.org/10.17605/OSF.IO/672EQ). The JBI three-step search process guided the development of the search strategy [13]. Searches were developed for databases: Ovid MEDLINE, CINAHL Complete (EBSCOhost), APA PsycInfo (EBSCOhost), and Embase (Elsevier) (Supplementary file 3). An extensive search of grey literature sources was undertaken (Appendix II).

### Inclusion and exclusion criteria

Literature was screened according to a pre-defined inclusion and exclusion criteria (Table 1). Literature published since 1 January 2014 was included to capture activity since the release of the *Review of Australian Government Health Workforce Programs*, which recommended longer term placements for health students in Australia, particularly in rural communities [15].

**Table 1:** Inclusion and exclusion criteria

	**Inclusion**	**Exclusion**
Population	Allied health, dentistry, medical, and nursing students completing an unpaid clinical placement as part of an undergraduate or postgraduate qualifying degree Allied health includes, but is not limited to, speech pathology, occupational therapy, optometry, podiatry, audiology, exercise physiology, medical imaging, psychology, physiotherapy, pharmacy, social work, chiropractors, dietitians, and paramedicine. [6]	Allied health, dentistry, medical, and nursing students completing a paid clinical placement as part of an undergraduate or postgraduate qualifying degree Allied health, dentistry, medical, and nursing postgraduate students undertaking an unpaid or paid clinical placement as part of an additional credential in addition to their qualification (e.g., Graduate Certificate or Diploma, Masters program, or Doctor of Philosophy) Allied health, dentistry, medical, and nursing student paid employment including, but not limited to, Registered Undergraduate Student of Nursing (RUSON) position, allied health assistants, or physician assistants
Concept	Peer-reviewed and non-peer-reviewed literature that examines the financial implications of undertaking unpaid clinical placements, including, but not limited to, observational studies, cross-sectional studies, qualitative studies, mixed-methods studies, economic studies, theses, and evaluation studies otherwise defined Unpaid is defined as a clinical placement without an allocated hourly wage, with or without a student scholarship or grant Financial implications includes but is not limited to, the direct and indirect costs of undertaking an unpaid clinical placement and financial-related stress	Commentaries, perspective pieces, editorials, media articles, and other opinion-based literature, will be excluded
Context	Literature examining unpaid clinical placements undertaken in the Australian setting and published since 1 January 2014	Studies not published in English

### Study selection and data extraction

Citations were imported into Covidence (Veritas Health Innovation, Melbourne, Australia). Titles and abstracts were screened independently by two reviewers. Full text review and data extraction was then undertaken. Findings were synthesised using a descriptive approach [13]. A quality assessment of included studies was undertaken using the JBI critical appraisal tools [16].

### Ethics

Ethics approval was not required for this review of the published research.

## Results

**Fig. 1:**
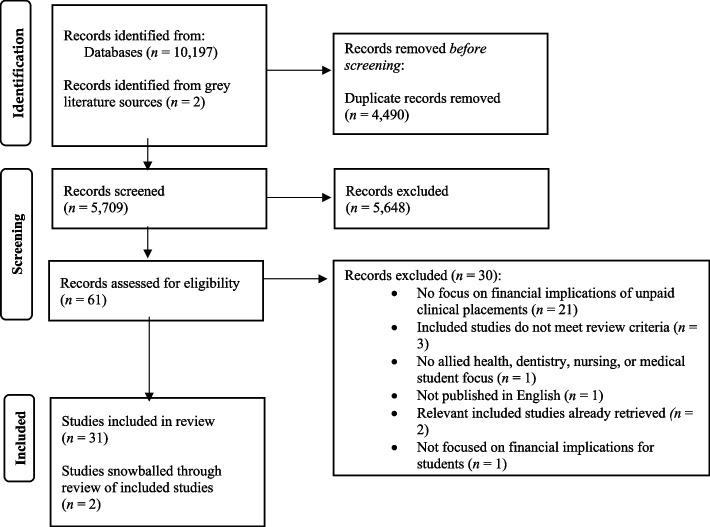
PRISMA Flow Diagram. *From:* Page MJ, McKenzie JE, Bossuyt PM, Boutron I, Hoffmann TC, Mulrow CD, et al. The PRISMA 2020 statement: an updated guideline for reporting systematic reviews. BMJ 2021;372:n71. 10.1136/bmj.n71

Searches returned 5,709 unique citations for title and abstract screening of which 5,648 were excluded Of 61 full text citations screened, 29 studies met the inclusion criteria. Reasons for exclusion were provided (Supplementary file 4). An additional two studies were retrieved from a review of grey literature databases and websites (Supplementary file 3) and two studies were identified from a review of the reference lists of included studies, yielding a total of 33 studies for inclusion (Fig. 1). Four studies were from one program of research which involved a national survey specific to social work students (National Study of Social Work Students) [17–20].

### Characteristics of included studies

Included studies (*n* = 33) were mostly descriptive (*n* = 32: cross-sectional studies *n* = 19; qualitative studies *n* = 9; mixed-method studies *n* = 4). One study (a longitudinal cohort study) involved an interventional component which evaluated student dietitians’ perceptions of a university-delivered psychoeducation service developed to support student ability to cope with placement stress (Table 2) [21]. Most studies focused on allied health professions (*n* = 12), followed by nursing students (*n* = 11), and medical students (*n* = 5). An additional five studies focused on multiple disciplines, including dentistry.

**Table 2:** Characteristics of included studies

Citation (Discipline)	Research aim	Study design type (methods; outcome measure)	Participant sample (female %; age)	Findings	Study limitations	Implications
Andrew et al. 2022 [36] *Nursing*	To explore the university experiences of undergraduate nursing students with family responsibilities	Qualitative study (interviews; experience)	T1 *n* = 29 (f 100%; mean 34 years); T2 *n* = 23 (f 100%; mean 34 years)	Two themes were identified, (1) family pressures, and (2) practicum poverty. Specifically, participants reflected on the difficulty in needing to balance paid work with their practicum which required them to work additional paid hours in the weeks before and after their practicum. This has flow on effects for managing study with participants reporting lower grades. As practicum lengths increased, so did the challenges of managing paid work with unpaid practicum (e.g., less able to use annual leave to support longer placements). Financial stress for participants' families, was inevitable	Study was limited to an exploration of heterosexual female student experience and may not be reflective of all nursing students	A need for a flexible and collaborative approach to support nursing students, particularly mature-aged nursing students, to undertake unpaid practicum is required. Revising financial supports for nursing students on placement is necessary to mitigate the financial stress experienced
Baglow & Gair 2019 [18] *Social Work*	To explore the impact of low or insufficient income on the lives and study experiences of Australian social work students	Cross-sectional study (survey—quantitative and qualitative responses; experience)	*n* = 2320 (f 87%; 69% 25 years and over)	Mature-aged students were more financially disadvantaged than younger students, with students receiving Aus Study most disadvantaged, with 36% of mature-aged students stating they had regularly gone without food or other necessities due to finances, 64% stating they did not have savings, and 61% stating they were not supported by parents, partners, or other family. A large proportion of mature-aged students stated they did not have enough money to pay for recommended texts (54%), were overtired from paid employment (47%), and had lower grades than expected (47%). Half of the students found increased financial burden due to placements	Study was largely descriptive and causal relationships between variables were not explored	Findings highlighted the need for greater financial support for mature-aged social work students, particularly with consideration of their family responsibilities. As a profession, social work is well placed to develop solutions to redress these issues
Baglow & Gair 2019 [19] *Social Work*	To detail preliminary findings around the impact of low levels of income on the lives and study success of social work students	Cross-sectional study (survey—quantitative and qualitative responses; experience)	*n* = 2320 (f 87%; 69% 25 years and over)	Of respondents, 47% received government financial assistance (e.g., youth allowance, Newstart allowance, Abstudy). The proportion of students reporting insufficient finances for necessities (e.g., food, clothing, accommodation, education resources, transport, and medication) was concerning	Survey questions may not allow student specific circumstances to be examined	Findings illuminated the issues of students balancing work, life, and study, including clinical placements
Birks et al. 2017 [43] *Nursing*	To examine nursing students' perceptions of block placements compared to a distributed model of clinical placement, on their learning experience	Qualitative (interviews and focus groups; perceptions)	*n* = 22 (f 91%; mean age 37.5 years)	Five themes were identified: (1) we're there to learn, (2) taking all that knowledge out and practicing it, (3) you actually feel a part of the team, (4) just prepare them for us coming, and (5) it's really individual. Specifically, for parents with young children, block placements were difficult and perceived to have a greater financial burden with accommodation and fuel costs, childcare costs, and loss of income	Study was confined to one state and limited by student availability to participate	Both block and distributed models of clinical placements have pros and cons which are dependent on the students' circumstance. This is important to consider for curriculum planning
Bradley, Bourke & Cosgrave 2020 [42] *Nursing and Allied Health*	To investigate the lived experiences of nursing and allied health students on placement in rural and regional Victoria	Qualitative study (semi-structured interviews; experience)	*n* = 18 (not reported)	Three enablers were identified positively impacting placement satisfaction: (1) enjoyment of rural environment and community, (2) working in a positive, friendly and supportive workplace, (3) exposure to broad clinical practice and enhanced learning opportunities. Five barriers were: (1) increased financial stress, (2) travel and accommodation challenges and concerns, (3) study-work-life balance and isolation issues, (4) encountering stressful work situations and/or personal events whilst on placement, and (5) communication issues with universities. Specifically, financial expenses due to placement caused stress and had negative consequences on employment including lost earnings, additional placement costs, vehicle maintenance, food, fuel, and uniform. Dual accommodation costs and managing the expectations of employers was challenging	Small sample	Greater financial support is required for health students on placement. UDRHs are well positioned to administer this to students
Brooke et al. 2020 [35] *Physiotherapy*	To determine perceived stress, burnout, and coping strategies in first year postgraduate pre-registration physiotherapy students	Cross-sectional study (survey—quantitative and qualitative responses; perceptions)	*n* = 38 (f 56%; median age 23 f, median age 26.5 m)	All participants reported a degree of stress and burnout. Analysis of open-ended questions identified seven themes: (1) curriculum coursework, (2) clinical placement, (3) transition periods, (4), work-life balance, (5) coping behaviours, (6) financial pressures, and (7) course survival tips. Participants reported difficulties whilst on placement attributed to travel and accommodation requirements and securing part-time employment	A methodological limitation was identified in a general lack of understanding of burnout as a concept. Study included a small sample size	Consistently high levels of stress amongst students were identified, including financial stress attributed to placements
Brosnan et al. 2016 [37] *Medicine*	To draw on the theory of Bourdieu to explore students who are the first in the family to attend university experiences of medical school	Qualitative study (interviews; experience)	*n* = 22 (f 77%; not reported)	Findings were reported against three concepts: (1) social capital, (2) economic capital, and (3) cultural capital. Perceived financial issues included the potential need to relocate for clinical placements	Small sample size	Admitting more students from diverse backgrounds to medicine is important, however, strategies to address challenges for such students is required
Cowan & Robinson 2023 [38] *Social Work*	To expand upon current knowledge of the impacts of lengthy unpaid block field placements on the physical, social, emotional, and financial well-being of social work students	Qualitative feminist paradigm (semi-structured interviews; experience)	*n* = 15 (f 100%; not reported)	Five themes were developed including 'caring as a female concern' which included three subthemes: (1) caring responsibilities: the definition of 'care', (2) planning and preparation for placement: covering care in absentia, and (3) juggling placement workload with life: choices within the boundaries of care	Data was collected pre-COVID-19	Social work cohorts largely comprise female students at a stage of life with caring responsibilities indicating current placement models are not fit for purpose. Issue of 'free degrees' not extending to allied health (e.g., as in Victoria). Need to examine different student cohorts and division of unpaid labour, including Aboriginal and Torres Strait Islander students. Calls for Australian Association of Social Workers to change their placement requirements
Crawford 2021 [48] *Nursing*	To identify factors and identify proactive approaches that impact the mental wellbeing and of mature-aged undergraduate university students, in and from, regional and remote Australia	Mixed-methods study (survey with open-ended questions and interviews; experience)	Overall study: *n* = 51 interviews (not reported); survey *n* = 1,783 (f 76%; not reported) Nursing participants *n* = 203 (not reported)	Specific to financial implications of unpaid placements, the need to relocate away from family, including children, for placements was a source of increased financial and emotional stress for mature-aged students	Potential for bias in recruitment and did not present findings by degree studied	Mature-aged students experience financial burdens when undertaking placements, particularly if they need to relocate for placements. Support for accommodation and travel is important
Elliot et al. 2023 [22] *Medicine*	To determine factors that impact overall medical student experience during rural placements	Cross-sectional study (survey; experience)	*n* = 107 (f 66.4%; not reported)	Participants had high levels of satisfaction with their rural/remote clinical placements. In terms of financial implications, 81.3% of students had access to accommodation, however, 33.3% of students stated they did not feel financially secure during their placement with a further 58.3% expressing dissatisfaction with support services for their families/spouse	Generalisability may be limited by selection and response bias	Overall students are satisfied with undertaking rural and remote placements. However, finances are a barrier that require targeted strategies
Gair & Baglow 2018 [17] *Social Work*	To explore the impact of low or insufficient income on the lives and study experiences of Australian social work students	Cross-sectional study (open-ended survey; experience)	*n* = 614 with qualitative responses (f 89%; 78% 25 years and over)	Six themes were presented: (1) a stressful balancing act, (2) significant sacrifices, (3) costs to health and wellbeing, (4) social work rhetoric and students' reality, (5) students forced to choose, and (6) suggestions for change. Specifically, most students highlighted the challenge of meeting financial obligations, including the costs of placement and factoring in paid work. Some participants shared that financial stress had caused them to take more extreme action (e.g., stealing food, illegally accessing books, concealing savings to maximise government benefits) and had repercussions for their health and wellbeing	Given focus of study, students with manageable financial circumstances may not have completed the survey	For social work students, managing lengthy unpaid field placements with work, family responsibilities, and study commitments is challenging and impacts their finances, family life, employment stability, health and wellbeing. Unity between universities and governments is required to redress this
Gair & Baglow 2018 [20] *Social Work*	To explore the impact of low income on social work students' daily lives and study access	Cross-sectional study (survey with open-ended questions; experience)	*n* = 2320 (f 87%; 69% 25 years and over)	Of respondents, 54% answered they had insufficient money for education resources. Themes developed included: (1) financial hardship impacts mental health and wellbeing, (2) forced decisions to gain necessities, (3) insufficient funds hinder mental health management, and (4) field placement increases ill-health/burnout. Respondents reinforced the financial and personal burden of placements as part of their studies	Study sample not representative of all students	Balancing financial hardship and study, including clinical placements, is a challenge for students. Reform is necessary, including increased financial support for students
Grant-Smith & de Zwaan 2019 [33] *Nursing*	To explore the financial impacts of clinical placement from the perspective of undergraduate nursing students in a large Australia university	Cross-sectional study (survey—quantitative and qualitative responses; experience)	*n* = 160 (f 91.4%; 45.6% between 21–30 years)	Findings were contained within four themes: (1) changes in personal financial circumstances and stress because of placement, (2) additional costs incurred as a result of placement, (3) other factors contributing to financial stress associated with placement, and (4) personal financial coping strategies and support seeking behaviours to manage the financial impact of placement. Specifically, the most reported impact of placements was on personal finances, followed by health and wellbeing. Associated costs included petrol, vehicle wear and tear, car servicing, road usage tolls, and parking costs. Other costs included childcare (including after school care)	Study relied on an opt in sampling approach	Findings highlighted the considerable financial stress experienced by nursing students due to clinical placements and highlights measures to address this, including more flexible approaches to placements
Hays, Devine and Glass 2022 [39] *Nursing*	To explore and describe students' experiences of studying nursing in the context of a satellite university campus located in a remote town	Qualitative study (semi-structured interviews; experience)	*n* = 9 (f 100%; 78% over 25 years)	Themes included: (1) remote campus experience, (2) learning experience, and (3) relationships and support. Specifically, feedback was received about the lack of support for remote students required to travel to attend clinical placements	Small sample size	Nursing students attending a remote university campus perceive their experience to differ to that of metropolitan nursing students
Hodge et al. 2021 [23] *Social Work*	To identify ways to better support the participation of women and diverse groups studying social work	Cross-sectional study (quantitative and qualitative responses; experience)	*n* = 61 (f 80%; 42.5% 18–24 years)	Themes included: (1) going from placement to work, (2) impacts on mental health and wellbeing, (3) the oppressive nature of placement, (4) self-care and maladaptive coping strategies, and (5) negative impacts on relationships and studies. The financial challenges associated with placements including needing to balance attendance with paid work, working long hours, having to quit paid employment due to placement attendance, and being unable to afford rent	Small sample size from a single university	Placements exacerbated financial hardship already experienced by students. Specific strategies to support students from low socio-economic backgrounds is required. Greater flexibility in placement hours and income during placement are potential strategies. Considering alternative field educations models is required
Jessup et al. 2022 [45] *Nursing, medical, and allied health*	To examine financial concern amongst health students during COVID-19, the financial implications of changes to planned rural or remote placements, and the impact of these factors on students' ability to undertake placements during the pandemic	Mixed-methods study (online survey, semi-structured interviews; perceptions, experience)	Online survey *n* = 1210 (f 84.5%; 59.7% under 25 years); semi-structured interviews *n* = 29 (not reported)	A key finding was the financial implications of placement changes during COVID-19 which included the positive financial implications of rural and remote placements being cancelled or changed with a closer placement which enabled students to maintain paid employment and save travel and accommodation expenses. Accommodation support from UDRHs was received. Negative financial implications included being unable to receive refunds from cancelled travel and accommodation and losing scholarships. Government payments also provided necessary financial stability	Self-selection and response bias from a convenience sample	Nursing, allied health and medical students with planned rural or remote placements during 2020 conveyed financial concerns due to placement changes. Targeted support for students attending rural or remote placements is required
Johnstone et al. 2016 [46] *Social Work*	To describe the prevalence of financial stress related to placements	Mixed-methods study (online survey, interviews and focus groups; experience)	Social work students *n* = 180, human service students (*n* = 34) (f 92%; not reported)	Due to studies, 57% of respondents had lost work shifts, 55% had cut work hours, and 35% had issues with their employer about not being available for work. Of respondents, 76% reported that field placement meant less time in paid employment which impacted their financial circumstances and led to stress. 19% reported changing course progression due to placement, and 20% had applied for financial support from their university. Solutions identified by students included: additional financial support/income, reduce placement requirements, revise process of negotiating placements, increase flexibility in how placements can be completed, and revise curriculum. Additional solutions identified by university field education and support staff included subsidies for transport costs, better communication around the hidden costs of placements, and exploring paid placement options	Low response rate (33%) and difficulty in obtaining a representative sample of respondents	Findings supported the likely association between unpaid placements and financial stress. Solutions for implementation were identified
King et al. 2016 [24] *Medicine*	To analyse rural clinical school students' perceptions of supports during extended blocks of clinical training	Cross-sectional study (survey; perceptions)	*n* = 454 (f 58.5%; not reported)	Most students felt well supported financially (60.5%)	Study did not ascertain the extent of financial support received by students	Overall, students had positive perceptions of rural clinical school support whilst on placement which is reassuring to validating the accessibility of the program
Kirkman et al. 2022 [44] *Optometry*	To explore factors which influence placement success and satisfaction from the perspective of optometry students	Qualitative study (focus groups; perspectives)	*n* = 42 (f 67%; not reported)	Four themes were identified: (1) changing identity, (2) dealing with complex dynamics and circumstances, (3) optometrist under instruction, and (4) rural practice is more rewarding. Specific to financial implications, relocation and transport costs were challenging for students, particularly those who were not able to attend placement where they had existing networks. Stress from reduced earnings and loss of employment income was discussed and particularly challenging for students with mortgages and partners. Costs associated with more remote placements were a disincentive	Study was a single-institution study	Extended clinical placements provided students with an opportunity to develop clinical skills. However, greater support for students undertaking rural placements in the form of financial assistance, is required
Koch et al. 2014 [25] *Nursing*	To describe the clinical experiences of nursing students and the diversity of characteristics that affect learning experience	Cross-sectional study (open-ended survey questions; experience)	*n* = 704 students (f 89%; not reported), *n* = 165 faculty members (89% f; not reported)	A theme 'surviving financially' described the financial strain of clinical placements from the perspectives of staff and students. Reasons identified included the inability for students to be employed, and the associated travel and accommodation costs for rural placements	Potential for responder bias	The financial strain of placements for nursing students was identified. Further exploration of this is required
Lane et al. 2020 [47] *Medicine*	To determine factors impacting the experiences of James Cook University medical students on solo placements in remote settings	Mixed-methods study (qualitative interviews and survey; experience)	*n* = 31 survey participants (f 74%; not reported); interview participants not reported	Two students responding to the open-ended survey questions acknowledged financial issues as a problem. From qualitative interviews, the additional financial costs of undertaking a remote placement (e.g., price of groceries in remote communities) were identified as challenging	Small sample of medical students from one University	Although solo remote placements have many benefits for medical students, there are financial implications (additional costs) associated with the remote context, that require consideration
Levett-Jones et al. 2015 [29] *Nursing*	To explore the concerns of first year Bachelor of Nursing Students from one Australian university as they prepare for their first clinical placement	Cross-sectional study (survey with open-ended questions; perceptions)	*n* = 262 (f 89%; mean 27 years)	Six themes were developed: (1) feeling nervous, (2) anxious and worried, (3) bullying and belonging, (4) practicalities, (5) patient safety and making mistakes, (6) working outside of my scope of practice. 12% of participants who responded to the open-ended questions expressed concerns around the costs of placement and location	Small sample size and feedback limited to written responses	Building a competent and confident nursing workforce is dependent on students who feel supported, including financially, and prepared for clinical placements
Luders et al. 2021 [26] *Nursing*	To evaluate Australian nursing students' views of placements at seven tertiary education institutions	Cross-sectional study (survey; perceptions)	*n* = 1263 (f 90%; 34.7% 26–40 years)	Overall, placements were positively rated. In terms of financial concerns, the loss of paid employment and costs of accommodation and transport were of concern	Low response rate (20%)	Further efforts and resourcing are required to support nursing student placements
Mortimer et al. 2019 [31] *Allied Health, Nursing, Midwifery, Medicine, Dentistry*	To compare a range of placement factors between health disciplines	Cross-sectional study (survey; experience)	*n* = 897 (f 75%; 64% 18–25 years)	Of respondents who had undertaken at least one rural placement (*n* = 565), non-medical students were provided with less support (including financial support or subsidised travel) than medical students in all domains, with the exception of cultural awareness training. Non-medical students were required to pay almost twice as much for their placement as medical students (mean total cost of placement of $1204.4 vs $641.2; *P* = 0.016). Part of this was attributed to the weekly cost of accommodation (mean: $117.7 non-medical vs $62.6 medical; *P* = 0.024). Of respondents who had not undertaken a rural placement (*n* = 332), 68 students had an opportunity to undertake a rural placement but chose not to due to social isolation (41%), lack of financial support (26%) and lack of organisational support (10%)	Possible selection bias	Rural placements were desired by students. However, uptake was affected by levels of support provided, including financial. It was also identified that non-medical students receive less support for rural placements than medical students which is an area requiring attention
Nedeljkovic et al. 2014 [27] *Psychology*	To develop and improve strategies ensuring the sustainable provision of university-based postgraduate psychology programs	Cross-sectional study (survey; experience)	*n* = 86 students (f 71%; 45% 25–29 years); *n* = 24 supervisors (not reported)	Most students reported being involved in the planning process of placement and most supervisors supported clinical placements as an important part of training. Financial difficulties, specifically, the hidden costs of placements, were raised in the student comments on the survey	Small sample size	A crisis in the provision of training and meeting workforce demand for psychologists is evident
Oke et al. 2022 [30] *Social Work*	To examine the experiences of social work students undertaking lengthy placements at a university in Australia	Cross-sectional study (survey with open-ended questions; experience)	*n* = 60 (f *n* = 48; *n* = 34 over 25 years)	79% of respondents stated they needed to work during their placement to meet the costs of necessities. Placements displaced paid employment for some students and many students had to alter their work schedules. Placement attendance created uncertainty within current employment	Small sample size	Requirements of lengthy unpaid clinical placements restrict how students can engage in the workforce
Podubinski et al. 2023 [61] *Nursing, Medical and Allied Health*	To explore self-reported mental health, stress and well-being concerns among allied health, nursing and medical students who completed a scheduled University Department of Rural Health facilitated placement in Australia between February and October 2020	Cross-sectional study (survey; experience)	*n* = 1315 (f 84%; mean 27 years)	Of respondents who completed a rural or remote placement, 55% expressed financial concerns during COVID-19. Multiple logistic regression models found clinical training, course progression, and financial concerns to be predictive of negative mental health	Difficult to determine response rate to survey and possibility of recall bias	Strategies to reduce financial stress, protect learning opportunities and increase connectedness for students undertaking a rural or remote placement, is required
Ross et al. 2022 [21] *Dietetics*	To evaluate student dietitians' perceptions of whether the program improved their ability to cope with practicum stressors	Longitudinal cohort study (repeat surveys, two cohorts; perceptions)	Survey one *n* = 84 (not reported), survey two *n* = 76 (not reported), survey three *n* = 94 (not reported)	Respondents identified the four most challenging aspects of clinical placement as constant assessment (56%), finances (40%), being away from usual supports (38%), and personality conflicts (35%). Students valued a psychoeducation resilience and wellbeing program delivered by a professional counselor at their university to manage their stress	Small sample and not representative of all students	Students find clinical placements to be stressful and challenging with added financial pressures. A psychoeducation service delivered by universities may support students in managing their stress
Saikal, Winona Pit & McCarthy 2020 [28] *Medicine*	To identify the predictors of well-being amongst a national sample of medical students on rural clinical placement	Cross-sectional study (survey; experience)	*n* = 644 (f 57%; 62% 25–34 years)	Respondents who felt supported financially (84% compared to 69%, *p* < 0.0001) and academically by their Rural Clinical School (87% compared to 34%, *p* < 0.0001), demonstrated a higher score of well-being compared with those who felt less supported	Findings did not distinguish between varying levels of wellbeing and possibility of selection bias	Findings add to the understanding between well-being and rural placements for medical students, with financial support a component of this
Salamonson et al. 2018 [40] *Nursing*	To explore the experiences of commencing first-year under-graduate nursing students who were studying full time while engaging in 20 or more hours of paid work each week	Qualitative exploratory study (semi-structured interviews; experience)	17 students (f *n* = 13; mean 33.4 years (19–49 years))	Four themes were identified: (1) work as a necessity…not a choice, (2) something's got to give, (3) it's a balancing act, and (4) being supported to work and study. In terms of placements, participants identified the expenses of buying items for clinical placement (e.g., uniform, shoes, equipment), and use of annual leave for mandatory clinical placements	Small sample	Paid work is not a choice for many nursing students, but a necessity. Given the financial burden of clinical placements, consideration is required as to how to balance unpaid placements with paid work to retain students
Smith et al. 2018 [32] *Nursing, Midwifery, Medical, Dentistry and Allied Health*	To understand the lived experience of students undertaking rural and remote placements	Cross-sectional study (open-ended survey questions; experience)	*n* = 3,204 students (f 79%; mean 26 years)	Three interrelated themes of ruralisation were identified: (1) preparation and support (including a sub-theme of financial support), (2) rural or remote health experience, and (3) rural lifestyle and socialisation. Difficulties with accommodation, internet, transport and financial support negatively impacted placement experience. Specifically, students expressed need for financial support and frustrating when they found out their placement did not qualify for rural funding support	Small sample size and confined to four open-ended survey questions	Cost of rural placements can be substantial for students, and add to anxiety. Although UDRHs provide some financial assistance, students identified issues around accessing other scholarships. Greater financial support for student placements in rural areas is required
Stuart & Gorman 2015 [41] *Nursing*	To explore the experiences of Indigenous health workers studying a Bachelor of Nursing degree	Qualitative interpretive study (in-depth interviews; experience)	*n* = 5 (f *n* = 3; age 36 to 52 years)	Six themes were identified: (1) recognition of prior skills, (2) issues pertaining to work and study, (3) support (financial, cultural, from academic staff, from Indigenous nurse academic, from family and peers), (4) racism (low expectations, stresses, confidence levels), (5) staying motivated, and (6) role models. Two sources of financial support were identified: National Indigenous Cadetship program and Indigenous scholarship. Without this support, participants acknowledged that they could not complete their degrees, particularly during clinical placements	Small sample size and only one Torres Strait Islander participant	Areas of support were identified that were necessary for Indigenous health workers to successfully graduate from a Bachelor of Nursing. The key area was financial support which can be met through scholarships
Usher et al. 2022 [34] *Nursing*	To investigate Australian nursing students' financial challenges related to mandatory work-integrated learning	Cross-sectional study (survey with open-ended questions; experience)	*n* = 2,359 (not reported)	Travel and transport: 68% of students travelled < 50 km to placement, whilst 15% travelled > 200 km. Most (72%) had a vehicle. More driving was required for placements with a lack of affordable accommodation. The times of public transport were not always convenient for placements. Accommodation: 16% accessed health service accommodation, whereas 33% had difficulties finding affordable accommodation. Respondents in urban areas had less trouble compared to regional areas (z = -3.78, *p* < 0.001 v. z = 2.88, *p* = 0.003). Weekly mean accommodation cost was $179 AUD. Employment: 83% of students were employed and most reported that placement affected this and resorted to using leave entitlements. Financial support: Centrelink was the most common financial support followed by a scholarship or allowance. 61% of students did not receive financial support. Financial support most often came from family, personal savings, working, and student loans. Home-related expenses: 75% rent/mortgage, 67% internet, 58% electricity, 41% gas, and other including childcare/school fees, transport costs, food, medical costs, and loan repayments. Placement expenses: the largest was lost wages of students and their partners who stepped into primary care responsibilities. Debt incurred: 35% incurred a mean debt of $1077 from placement in the form of a family loan, credit card debt, personal loan, or other. Those who travelled further, had more debt. Strategies to reduce costs: included selecting locations with low or free parking, close to family/friends or travelling with other students	Unable to determine causality due to study design	Not only are the costs due to clinical placements for nursing students under-studied but are also significant in terms of loss of earnings and placement-related expenses. There is a need for universities and placement providers to reduce these costs

### Reliance on self-reported measures of financial implications

All studies relied on self-reported measures to describe the financial implications of unpaid clinical placements. Cross-sectional survey studies used bespoke open and close-ended questions to examine dimensions of self-reported student placement experiences and perceptions including; student allowances and government assistance received, hours of paid work, reasons for engaging in paid work, other support received (e.g., family financial support), managing family responsibilities, impact of reduced income on study and life, income, savings [17, 18, 22–30], rural health placement experience [31, 32], and financial wellbeing and needs [33, 34]. Some studies also incorporated the use of validated questionnaires into surveys, including the Coping Self-Efficacy Scale, and the Maslach Burnout Inventory-General Survey for Students, to examine other variables of relevance to financial implications identified [35]. Similarly, the longitudinal cohort study relied on self-reported measures of placement experience using open and closed-ended survey questions over time [21].

Qualitative studies expanded on the implications of unpaid clinical placements by exploring self-reported measures such as participant experiences [36–42], perceptions [43], and perspectives of factors influencing placement outcomes which included financial implications [44]. Similarly, mixed-methods studies used a combination of open and closed questions to examine dimensions of self-reported placement experience, financial position, and employment, in addition to qualitative interviews examining participant experience [45–47].

### Costs of unpaid clinical placements for students

The types of costs incurred by students due to unpaid clinical placements were identified by studies (Table 3). These were categorised as direct, indirect, and hidden costs. Direct costs were considered expenses attributed to a clinical placement that were necessary to complete a placement and included relocation, travel, uniforms, and equipment. One study quantified the weight of direct costs varied across disciplines and found that non-medical students (including nursing and allied health students), were required to pay almost twice as much for their placement as medical students (mean total cost of placement of $1204.4 vs $641.2; *P* = 0.016), partly attributed to the weekly out-of-pocket cost of accommodation (mean: $117.7 non-medical vs $62.6 medical; *P* = 0.024) [31]. This was attributed to medical students receiving greater financial support for accommodation [31].

**Table 3:** Direct, indirect, and hidden costs of unpaid clinical placements for students

	**Examples identified by included studies**
**Direct costs**	*Accommodation* required for placements away from home which may be partly subsidised by a Rural Clinical School (medical students) or University Department of Rural Health (allied health and nursing), if a rural or remote placement [22, 26, 32–34, 42, 43, 45].
	*Travel* including use of private vehicle, wear and tear, fuel, or public transport [22, 30, 32–36, 38, 39, 42, 45, 46].
	*Uniforms* including institutional uniform and occupational footwear [33, 40, 42].
	*Equipment* including stethoscope, fob watch, and stationary [33, 40].
**Indirect costs**	*Loss of income* or *reduced earnings* from employment [26, 30, 33, 34, 36, 38, 42, 43].
	*Childcare* including after-school childcare to accommodate placement hours [33, 38, 43].
	*Rent* or *mortgage* paid on primary place of residence [17, 34, 42].
	*Inflated costs of groceries* purchased during rural or remote placements [47].
	*Interest payable* on debts obtained to support student during placement [34].
**Hidden costs**	*Parking fees* including hospital parking fees [27, 30, 34].
	*Road tolls* payable for commute to metropolitan placements [33].

Indirect costs include expenses necessary to support a student to complete an unpaid clinical placement that may not be directly related to the clinical placement itself. Examples included a loss of income or reduced earnings, childcare, and interest payable on debts to support placement (Table 3). Hidden costs were expenses that were not foreseeable by students but accumulated throughout clinical placements and included parking fees and road tolls (Table 3).

## Implications of costs for students

Costs associated with unpaid clinical placements exacerbated existing financial hardship [23], and the financial burden experienced by students, particularly those on longer placements (Table 4) [30, 36, 46]. Students from specific cohorts (those with family or caring responsibilities [18, 30, 36, 38, 43], with partners working in low-paid occupations [36], from low-socio-economic backgrounds or rural/remote backgrounds, Aboriginal and/or Torres Strait Islander [41], and mature-aged students with children [18, 48]) were identified as particularly vulnerable to financial hardship.

**Table 4:** Key concepts of implications of placement costs for students

Concept	Description
Balancing paid work and unpaid placement	Needing to balance paid work with unpaid clinical placement was difficult [23, 25, 26, 33, 46]. Challenges included navigating employer expectations [30, 42], needing to work extra hours before or after placement, including on weekends [41], and experiencing a loss or reduced income [34, 36, 38, 42]. Individual strategies to manage this included the use of annual leave to support unpaid placement [40, 45], and preference for casual employment for flexibility with placement periods [36].
Travelling to a clinical placement	The need to travel long distances from participant home addresses and associated financial burden of this including the need to source accommodation [34, 42], was a recurrent issue identified in studies [25, 26, 29, 30, 36, 44, 45, 48]. For disciplines such as nursing, it was highlighted that placement allocation was beyond the control of students which was particularly stressful [36]. Further, given the location of placements and placement hours, navigating public transport was problematic for some students [26, 34], and not an option for others, including remote students travelling to metropolitan placements [34, 37] and metropolitan students traveling to rural and remote communities
Going without necessities	Studies identified that due to the financial implications of unpaid clinical placements, students reported going without food, other necessities (including health care), and educational resources [17], or reducing their expenditure on these items [33].
Managing family responsibilities	For students with families, balancing unpaid placements with family responsibilities was challenging [17, 18, 23, 26, 30, 33, 48]. Additional childcare costs and the difficulty in managing irregular shift work hours around the needs of children, were frequently cited [17, 20, 36, 38]. Coordinating this with partner work schedules was problematic, particularly for students with partners working in ‘fly-in-fly-out’ positions [36].
Feeling run down	Generally, the financial impacts of placement had impacts on health and wellbeing, including deterioration of mental health [17, 20, 23, 33, 46]. This was also exacerbated for students working additional hours of paid employment [25]. In addition to the financial stress of unpaid placements, students were also required to complete assessments for coursework concurrently with placements which exacerbated the feeling of being run down [17, 36].
Needing financial support	Given the direct, indirect, and hidden costs of placements, studies consistently identified the need and benefits of additional financial support for students undertaking unpaid clinical placements, particularly those from more vulnerable cohorts [19, 32, 39, 41, 45].
Impacts on learning	Some studies captured the impacts of financial stress experienced due to unpaid clinical placements on coursework [17, 23]. Key issues included students reporting lower grades, skipping classes to attend paid work, needing to defer a course or reduce course work [17, 23].

Placement costs were identified as a disincentive for students from metropolitan areas to gain rural or remote clinical experience [32, 44]. For example, a mixed-methods study of nursing, allied health, and medical student placement experiences during COVID-19 reported positive financial impacts, such as saving on travel and accommodation, when rural or remote placements were cancelled or changed to metropolitan locations due to the pandemic [45]. Further, a national survey of medical and non-medical students who were members of the National Rural Health Student Network (*n* = 897) found that 26.5% of respondents chose to avoid a rural placement due to a lack of financial support [31].

Aside from some student disciplines (i.e., medicine) receiving subsidies for placements [22, 28], unpaid placements generally required students to rely on other sources of financial support. One study identified that 35% of survey respondents (nursing students) had incurred debt from their clinical placement, either from friends of family, or loans, with students who traveled further (101–200 km and over 200 km) incurring more debt [34]. Another survey study identified that 56% of undergraduate and 56% of post-graduate social work student respondents did not receive financial support from family [17]. Relying on employment income was frequently cited as problematic given the need to have time off work to attend placements, work additional hours in the period before and after placements, and use of annual leave to support an unpaid placement [23, 25, 26, 33, 40, 45, 46].

Financial stress had flow on effects for student health and wellbeing [20]. One study found that medical students who felt financially and academically supported by their Rural Clinical School, had a higher score of well-being, when compared to students who did not feel supported [28]. Financial stress was also identified as a contributor to student attrition rates, burn out, poor academic performance and food insecurity [17, 35]. Medical students were not exempt from this, with one national survey identifying that 33% of respondents (*n* = 107) did not feel financially secure during their unpaid placement [22].

## An urgent need for targeted strategies to redress

Most studies identified the need for immediate strategies to alleviate the financial burden of unpaid clinical placements for students, particularly for students undertaking rural and remote placements [32], and from otherwise vulnerable backgrounds. There was evidence of strategies activated at a student level. For example, one study which involved 160 nursing students identified frequently applied strategies as: saving prior to placement (*n* = 49, 31%), working more before placement in paid employment (*n* = 24, 15%), reducing spending on food (*n* = 20, 12%), and reducing expenditure on non-essential items (*n* = 16, 10%) [31]. However, unanimous support was provided by studies for the need for collaborative efforts to develop and implement additional strategies (Table 5). These include revising existing financial supports, placement models across disciplines, and university placement procedures, and implementing placement-specific support services.

**Table 5:** Strategies to alleviate the financial burden of unpaid clinical placements

Strategy	Description
Revise existing financial supports and consider additional supports	Revise existing financial supports for students across disciplines to examine whether they are sufficient (e.g., accommodation and travel support provided by University Departments of Rural Health and Rural Clinical Schools, funded by the Australian Government’s Rural Health Multidisciplinary Training program), particularly for mature-aged students, students with family responsibilities, from low-socioeconomic backgrounds, Aboriginal and/or Torres Strait Islander students, and rural or remote students undertaking a metropolitan placement, and metropolitan students undertaking a rural or remote placement [18, 19, 23, 24, 32, 34, 36, 38, 39, 41, 45, 46]. Consider gaps in support available for students
Revise placement models across disciplines	Consider different models of placements for different student cohorts (e.g., block, distributed), including more flexible placement models (e.g., adjustment of placement hours) to accommodate the division of unpaid labour (e.g., primary carers for children) and paid labour [19, 23, 33, 38, 40, 43, 46].
Revise university placement procedures	Revise university rules around students undertaking paid work during placement periods and process for negotiating placements [23, 27, 46]. Giving students a choice to locate placements near family or friends or with other students could ease the financial burden [34]. Providing students with sufficient notice of their placement (including facility and work schedule) and avoiding the delivery of coursework and assessments concurrently, was also supported [36].
Implement placement-specific support services	Implement support services to help students manage stress associated with clinical placements [21, 46].

## Quality assessment and study limitations

All studies relied on self-reported data. Selection and reporting bias were cited as key methodological limitations [19], in addition to small sample sizes that may not be representative of all student cohorts. Given study designs used, studies did not control for confounders or examine causal relationships between variables which was identified as a limitation [18]. Cross-sectional studies cited potential bias in the development of survey instruments [17]. Tables 6, 7 and 8.

**Table 6:** Quality assessment of cross-sectional studies

Citation	Were the criteria for inclusion in the sample clearly defined?	Were the study subjects and the setting described in detail?	Was the exposure measured in a valid and reliable way?	Were objective, standard criteria used for measurement of the condition?	Were confounding factors identified?	Were strategies to deal with the confounding factors stated?	Were the outcomes measured in a valid and reliable way?	Was appropriate statistical analysis used?
Baglow & Gair 2019 [17]	Y	Y	Y	Y	NA	NA	Y	Y
Baglow & Gair 2019 [18]	Y	Y	Y	Y	NA	NA	Y	Y
Brooke et al. 2020 [35]	Y	Y	Y	Y	NA	NA	Y	Y
Elliot et al. 2023 [22]	Y	Y	Y	Y	NA	NA	Y	Y
Gair & Baglow 2018 [17]	Y	Y	Y	Y	NA	NA	Y	Y
Grant-Smith & deZwaan 2019 [33]	Y	Y	Y	Y	NA	NA	Y	Y
Hodge et al. 2021 [23]	Y	Y	Y	Y	NA	NA	Y	Y
King et al. 2016 [24]	Y	N	Y	Y	NA	NA	Y	Y
Koch et al. 2014 [25]	Y	Y	Y	Y	NA	NA	Y	Y
Luders et al. 2021 [26]	Y	Y	Y	Y	NA	NA	Y	Y
Mortimer et al. 2019 [31]	Y	Y	Y	Y	NA	NA	Y	Y
Nedeljkovic et al. 2014 [27]	Y	Y	Y	Y	NA	NA	Y	Y
Podubinski et al. 2023 [61]	Y	Y	Y	Y	NA	NA	Y	Y
Saikal, Winona Pit & McCarthy 2020 [28]	Y	Y	Y	Y	NA	NA	Y	Y
Smith et al. 2018 [32]	Y	Y	Y	Y	NA	NA	Y	NA
Usher et al. 2022 [34]	Y	Y	Y	Y	NA	NA	Y	Y
Levett-Jones et al. 2015 [29]	Y	Y	Y	Y	NA	NA	Y	NA
Gair & Baglow 2018 [20]	Y	Y	Y	Y	NA	NA	Y	NA
Oke et al. 2022 [30]	Y	Y	Y	Y	NA	NA	Y	NA
Jessup et al. 2022 [45]	Y	Y	Y	Y	NA	NA	Y	Y
Johnstone et al. 2016 [46]	Y	Y	Y	Y	NA	NA	Y	Y
Lane et al. 2020 [47]	Y	Y	Y	Y	NA	NA	Y	Y
Crawford 2021 [48]*	Y	Y	Y	Y	NA	NA	Y	Y

**^***Y*  Yes, *N*  No, and *NA* Not applicable

^*^Study not peer-reviewed

**Table 7:** Quality assessment of cohort studies

Citation	Were the two groups similar and recruited from the same population?	Were the exposures measured similarly to assign people to both exposed and unexposed groups?	Was the exposure measured in a valid and reliable way?	Were confounding factors identified?	Were strategies to deal with confounding factors stated?	Were the groups/participants free of the outcome at the start of the study (or at the moment of exposure)?	Were the outcomes measured in a valid and reliable way?	Was the follow up time reported and sufficient to be long enough for outcomes to occur?	Was follow up complete, and if not, were the reasons to loss to follow up described and explored?	Were strategies to address incomplete follow up utilised?	Was appropriate statistical analysis used?
Ross et al. 2022 [21]	Y	N/A	Y	N	N/A	Y	Y	Y	N	NA	Y

**^***Y*  Yes, *N*  No, and *NA*  Not applicable

**Table 8:** Quality assessment of qualitative studies

Citation	Is there congruity between the state philosophical perspective and the research methodology?	Is there congruity between the research methodology and the research question or objectives?	Is there congruity between the research methodology and the methods used to collect data?	Is there congruity between the research methodology and the representation and analysis of data?	Is there congruity between the research methodology and the interpretation of results?	Is there a statement locating the researcher culturally or theoretically?	Is the influence of the researcher on the research, and vice-versa, addressed?	Are participants, and their voices, adequately represented?	Is the research ethical according to current criteria or for recent studies, and is there evidence of ethical approval by an appropriate body?	Do conclusions drawn in the research report flow from the analysis, or interpretation, of the data?
Andrew et al. 2022 [36]	Y	Y	Y	Y	Y	N	N	Y	Y	Y
Birks et al. 2017 [43]	Y	Y	Y	Y	Y	N	N	Y	Y	Y
Brosnan et al. 2016 [37]	Y	Y	Y	Y	Y	N	N	Y	Y	Y
Cowan & Robinson 2023 [38]	Y	Y	Y	Y	Y	N	Y	Y	Y	Y
Hays, Devine & Glass 2022 [39]	Y	Y	Y	Y	Y	N	Y	Y	Y	Y
Kirkman et al. 2022 [44]	Y	Y	Y	Y	Y	N	N	Y	Y	Y
Salamonson et al. 2018 [40]	Y	Y	Y	Y	Y	N	N	Y	Y	Y
Stuart & Gorman 2015 [41]	N	Y	Y	Y	Y	N	N	Y	Y	Y
Bradley, Bourke & Cosgrove 2020 [42]	N	Y	Y	Y	Y	N	N	Y	Y	Y
Jessup et al. 2022 [45]	N	Y	Y	Y	Y	N	N	Y	Y	Y
Johnstone et al. 2016 [46]	N	Y	Y	Y	Y	N	N	Y	Y	Y
Lane et al. 2020 [47]	N	Y	Y	Y	Y	Y	N	Y	Y	Y
Crawford 2021^a^ ^48^	Y	Y	Y	Y	Y	Y	Y	Y	Y	Y

**^***Y*  Yes, *N* No, and *NA*  Not applicable

^*a*^*Study not peer-reviewed*

## Discussion

The financial implications of unpaid clinical placements for allied health, dentistry, medical, and nursing students in Australia, are well demonstrated in the research literature. Financial implications have been examined using self-reported measures from surveys and qualitative research. Novel review findings include the categorisation of the direct, indirect, and hidden costs of unpaid clinical placements, implications of these for students, and potential strategies to redress.

What is concerning is that the adverse financial implications of unpaid clinical placements for students (otherwise known as ‘placement poverty’) is not a new issue identified in research. Despite concerns over future workforce sustainability, very little progress has been made to redress this issue and move beyond the use of self-reported measures for research gain. Even prior to 2014, there is evidence of research considering the financial implications of unpaid clinical placements for health students[49–51]. This includes a study undertaken by Kenny and colleagues (2011) which identified financial constraints as a key driver of mature-aged nursing student attrition [52]. Given only one retrieved study included an interventional component (a psychoeducational program to support dietetics students experiencing financial difficulties during placement) [21], research efforts have not shifted post-2014. Efforts have been focused on describing inequity and identifying potential solutions rather than implementing strategies to alleviate the financial implications of unpaid clinical placements – a gap in activity identified by this review. Action is necessary to redress this inertia and translate developed strategies into research, policy, and practice. Without well-supported students, and subsequently confident and competent health professionals, the Australian health system could face dire consequences that ultimately impact on patient care, particularly in rural communities [53].

Beyond research, there has been growing debate in the Australian media, advocacy by peak health associations, and universities (e.g., Australian Universities Accord) [54], and discussion in government to develop solutions. The result is a means-tested placement support program for nursing, social work, and education students announced as part of the 2024/2025 Australian Government budget (up to $319.50 payment per week of placement) [55]. In addition, the Australian Government has also changed rules of the HECS-HELP student debt to ensure indexing is in line with wage growth rather than the consumer price index which is elevated when compared to previous years [56]. Although these initiatives are welcomed, there are some key issues identified by this review which require immediate consideration as these policies are implemented. Most notable is the exclusion of allied health student cohorts beyond social work. On this basis, two recommendations are provided based on review findings.

## Scope the needs of different student cohorts

It is important to consider the diversity of student needs across disciplines, including students with family responsibilities, from rural or remote communities, from low-socio-economic backgrounds, of Aboriginal and/or Torres Strait Islander background, and to evaluate interventions with this in mind. The recently announced placement support payment in Australia [55] should be examined to determine sufficiency to alleviate financial stress across identified nursing and social work student cohorts,particularly students completing regional, rural or remote placements Examining what proportion of students would be eligible for means tested support payments and rates of attrition, is key to this. This is important given the known impacts of stress on student wellbeing, impacts on academic performance, and documented rigidity of university placement processes [19]. For example, research undertaken with students has identified that nursing students have inadequate sleep [57] and are at risk for self-medicating due to stress [58], and a proportion of medical students are at risk for depression [59]. Alleviating financial stress on students is critical to ensuring a well-prepared graduate workforce.

## Develop, implement, and evaluate a suite of strategies

There is a need to develop a suite of strategies to alleviate the direct, indirect, and hidden costs of unpaid clinical placements for allied health, medical, and nursing students; or we risk avoidable health system deterioration. To develop these strategies, it is imperative to understand barriers to enacting reforms. To achieve this, it is important for policymakers to collaborate with universities, health services, communities, peak professional bodies, and placement host organisations, and leverage from existing supports (e.g., accommodation support provided by University Departments of Health for rural placements, funded through the Australian Government’s Rural Health Multidisciplinary Training program). Understanding existing models of clinical placements (e.g., mandatory regional, rural, or remote placements) is a necessary first step. This will be important to developing hybrid models of clinical placements which allow students to maintain existing employment (e.g., undertake placement part-time) or gain industry relevant employment that is recognised as equivocal to clinical placement. Understanding graduate outcomes of students who engage with industry relevant paid employment (e.g., Registered Undergraduate Student of Nursing), with those who do not, requires attention [60]. Examining models across industries is a useful starting point; for example fully-funded bonded training programs requiring graduates to work with organisations for a few years post-graduation. A framework for evaluation should be established from the outset to build the evidence base as to what works, for whom, and in what context. This includes embedding financial measures, such as those identified in this review, in existing programs of research examining student outcomes.

## Limitations

This review was limited to Australian research to map knowledge within the Australian context to inform policy, research, and practice. It is likely that the direct, indirect, and hidden costs of unpaid clinical placements are shared by allied health, dentistry, medical, and nursing students internationally. However, an international review would be valuable to examine evidence of solutions, programs, and strategies to financially support clinical placements for nursing, allied health, medical and dentistry students across a range of contexts and clinical settings.

## Conclusions

The financial implications of unpaid clinical placements for allied health, medical, and nursing students in Australia are well demonstrated and have significant implications for the future of Australia’s health workforce and health system. Research findings have been consistent over the past decade in advocating for greater financial support for students undertaking unpaid clinical placements. This includes the need for greater flexibility of placement models, so the indirect costs of placements are mitigated. Collaboration between governments, universities, peak professional bodies, and placement host organisations is imperative to implementing and evaluating a suite of strategies to redress the financial burden experienced by students; and thus secure the future of Australia’s health workforce.

## Supplementary Information

Supplementary file 1. PRISMA -ScR checklist

Supplementary file 2. Electronic searches

Supplementary file 3. Grey literature searches

Supplementary file 4. Excluded studies

## Data Availability

No datasets were generated or analysed during the current study.
