# Supplementary material for: Financial implications of unpaid clinical placements for allied health, dentistry, medical, and nursing students in Australia: a scoping review with recommendations for policy, research, and practice
Source: BMC Health Serv Res. 2024 Nov 15;24:1407. doi: 10.1186/s12913-024-11888-y (PMC11566465; doi:10.1186/s12913-024-11888-y)
Supplement: Supplementary file 2 — Electronic searches [file 12913_2024_11888_MOESM2_ESM.docx]

**Supplementary file 2. Electronic searches**

| **Database** | **Citations retrieved** |
| --- | --- |
| Ovid MEDLINE | 3,136 |
| CINAHL (EBSCOhost) | 2,520 |
| APA PsycInfo (EBSCOhost) | 1,005 |
| Embase (Elsevier) | 3,536 |
| Total | 10,197 |
| Duplicate papers | 4,490 |
| Total left to screen | 5,648 |

Database searches were conducted on the 21 February 2023

Database: Ovid MEDLINE® Epub Ahead of Print, In-Process & Other Non-Indexed Citations, Ovid MEDLINE(R) Daily, Ovid MEDLINE and Versions(R)

| Query | Search terms |
| --- | --- |
| 1 | exp Health Occupations/ |
| 2 | exp Health Workforce/ |
| 3 | exp Health Personnel/ |
| 4 | (physiotherap* or speech path* or diet* or occupational therap* or podiatr* or social work* or psych* or pharm* or chiropractor* or audiolog* or allied health* or exercise physio* or medical imag* or optometr* or dentist* or paramed*).mp. |
| 5 | (nurs* or midwi*).mp. |
| 6 | (medic*).mp. |
| 7 | OR/1-6 |
| 8 | (student* or undergrad*).mp. |
| 9 | exp Students, Health Occupations/ |
| 10 | OR/8-9 |
| 11 | exp clinical clerkship/ |
| 12 | (clerkship* OR placement* OR fieldwork* OR intern* OR WIL OR work integrated learning OR preceptor* OR educat* or prac*).mp. |
| 13 | OR/11-12 |
| 14 | exp Australia/ |
| 15 | (Austral* or Tasmania* or Victoria* or New South Wales or Queensland or Northern Territor*).mp. |
| 16 | OR/14-15 |
| 17 | 7 AND 10 AND 13 AND 16 |
| 18 | Limit 17 to yr=”2014-Current” |

Database: CINAHL Complete (EBSCOhost)

| Query | Search terms |
| --- | --- |
| S1 | (MH " Health Occupations+") |
| S2 | (MH "Health Labor Supply+") |
| S3 | (MH "Health Personnel+") |
| S4 | TI ((physiotherap* OR "speech path*" OR diet* OR "occupational therap*" OR podiatr* OR "social work*" OR psych* OR pharm* OR chiropractor* OR audiolog* OR "allied health*" OR "exercise physio*" OR "medical imag*" OR optometr* OR dentist* OR paramed*)) OR AB ((physiotherap* OR "speech path*" OR diet* OR "occupational therap*" OR podiatr* OR "social work*" OR psych* OR pharm* OR chiropractor* OR audiolog* OR "allied health*" OR "exercise physio*" OR "medical imag*" OR optometr* OR dentist* OR paramed*)) |
| S5 | TI ((nurs* OR midwi*)) OR AB ((nurs* OR midwi*)) |
| S6 | TI (medic*) OR AB (medic*) |
| S7 | S1 OR S2 OR S3 OR S4 OR S5 OR S6 |
| S8 | TI ((student* OR undergrad*)) OR AB ((student* OR undergrad*)) |
| S9 | (MH "Students, Health Occupations+") |
| S10 | S8 OR S9 |
| S11 | (MH "Student Placement+") |
| S12 | TI ((clerkship* OR placement* OR fieldwork* OR intern* OR WIL OR "work integrated learning" OR preceptor* OR educat* OR prac*)) OR AB ((clerkship* OR placement* OR fieldwork* OR intern* OR WIL OR "work integrated learning" OR preceptor* OR educat* OR prac*)) |
| S13 | S11 OR S12 |
| S14 | (MH Australia+) |
| S15 | TI ((Austral* OR Tasmania* OR Victoria* OR "New South Wales" OR Queensland OR "Northern Territor*")) OR AB ((Austral* OR Tasmania* OR Victoria* OR "New South Wales" OR Queensland OR "Northern Territor*")) |
| S16 | S14 OR S15 |
| S17 | S7 AND S10 AND S13 AND S16 |
| S18 | Limit 17 to yr=”2014-Current”" |

Database: APA PsycInfo (EBSCOhost)

| Query | Search terms |
| --- | --- |
| S1 | (DE "Health Personnel") |
| S2 | TI ((physiotherap* OR "speech path*" OR diet* OR "occupational therap*" OR podiatr* OR "social work*" OR psych* OR pharm* OR chiropractor* OR audiolog* OR "allied health*" OR "exercise physio*" OR "medical imag*" OR optometr* OR dentist* OR paramed*)) OR AB ((physiotherap* OR "speech path*" OR diet* OR "occupational therap*" OR podiatr* OR "social work*" OR psych* OR pharm* OR chiropractor* OR audiolog* OR "allied health*" OR "exercise physio*" OR "medical imag*" OR optometr* OR dentist* OR paramed*)) |
| S3 | TI ((nurs* OR midwi*)) OR AB ((nurs* OR midwi*)) |
| S4 | TI (medic*) OR AB (medic*) |
| S5 | S1 OR S2 OR S3 OR S4 |
| S6 | TI ((student* OR undergrad*)) OR AB ((student* OR undergrad*)) |
| S7 | (DE Students) |
| S8 | S6 OR S7 |
| S9 | (DE “Educational placement”) |
| S10 | TI ((clerkship* OR placement* OR fieldwork* OR intern* OR WIL OR "work integrated learning" OR preceptor* OR educat* OR prac*)) OR AB ((clerkship* OR placement* OR fieldwork* OR intern* OR WIL OR "work integrated learning" OR preceptor* OR educat* OR prac*)) |
| S11 | S9 OR S10 |
| S12 | TI ((Austral* OR Tasmania* OR Victoria* OR "New South Wales" OR Queensland OR "Northern Territor*")) OR AB ((Austral* OR Tasmania* OR Victoria* OR "New South Wales" OR Queensland OR "Northern Territor*")) |
| S13 | S5 AND S8 AND S11 AND S12 |
| S14 | Limit 13 to yr=”2014-Current”" |

Database: Embase (Elsevier)

| Query | Search terms |
| --- | --- |
| 1 | ‘occupation’/exp |
| 2 | ‘health workforce’/exp |
| 3 | ‘health care personnel’/exp |
| 4 | (physiotherap* OR "speech path*" OR diet* OR "occupational therap*" OR podiatr* OR "social work*" OR psych* OR pharm* OR chiropractor* OR audiolog* OR "allied health*" OR "exercise physio*" OR "medical imag*" OR optometr* OR dentist* OR paramed*):ti,ab,kw |
| 5 | (nurs* OR midwi*):ti,ab,kw |
| 6 | (medic*):ti,ab,kw |
| 7 | #1 OR #2 OR #3 OR #4 OR #5 OR #6 |
| 8 | (student* OR undergrad*):ti,ab,kw |
| 9 | ‘health student’/exp |
| 10 | #8 OR #9 |
| 11 | ‘clinical education’/exp |
| 12 | (clerkship* OR placement* OR fieldwork* OR intern* OR WIL OR "work integrated learning" OR preceptor* OR educat* OR prac*):ti,ab,kw |
| 13 | #11 OR #12 |
| 14 | ‘Australia’/exp |
| 15 | (Austral* OR Tasmania* OR Victoria* OR "New South Wales" OR Queensland OR "Northern Territor*"):ti,ab,kw |
| 16 | #14 OR #15 |
| 17 | #7 AND #10 AND #13 AND #16 |
| 18 | #17 AND [2014]2024]/py |
