# Supplementary material for: Financial implications of unpaid clinical placements for allied health, dentistry, medical, and nursing students in Australia: a scoping review with recommendations for policy, research, and practice
Source: BMC Health Serv Res. 2024 Nov 15;24:1407. doi: 10.1186/s12913-024-11888-y (PMC11566465; doi:10.1186/s12913-024-11888-y)
Supplement: Supplementary file 3 — Grey literature searches [file 12913_2024_11888_MOESM3_ESM.docx]

**Supplementary file 3. Grey literature sources**

| **Australia** | |
| --- | --- |
| Australian College of Nursing | https://www.acn.edu.au/ |
| Australian Medical Association | https://www.ama.com.au/ |
| Australian Nursing & Midwifery Journal | https://anmj.org.au/ |
| Congress of Aboriginal and Torres Strait Islander Nurses and Midwives (CATSINaM) | https://catsinam.org.au/ |
| Australian Government Department of Health | https://www.health.gov.au/ |
| Services for Australian Rural and Remote Allied Health | https://sarrah.org.au/ |
| National Rural Health Alliance | https://www.ruralhealth.org.au/ |
| Australian Medical Students’ Association | https://www.amsa.org.au/Web/Web/Home.aspx |
| National Rural Health Student Network | https://nrhsn.org.au/ |
| Allied Health Professionals Australia | https://ahpa.com.au/ |
| TROVE | https://trove.nla.gov.au/ |
| OAIster | https://oaister.on.worldcat.org/discovery |
| Google | www.google.com |
